# Spectroscopic, Thermal, and Antimicrobial Studies of Co(II), Ni(II), Cu(II), and Zn(II) Complexes Derived from Bidentate Ligands Containing N and S Donor Atoms

**DOI:** 10.1155/2012/729708

**Published:** 2012-11-22

**Authors:** Kiran Singh, Yogender Kumar, Parvesh Puri, Gulab Singh

**Affiliations:** ^1^Department of Chemistry, Kurukshetra University, Kurukshetra 136 119, India; ^2^Department of Microbiology, Kurukshetra University, Kurukshetra 136 119, India

## Abstract

Two new heterocyclic Schiff bases of 4-amino-5-mercapto-3-H/propyl-1,2,4-triazole and 5-nitrofurfuraldehyde [HL^1-2^] and their cobalt, nickel, copper, and zinc complexes have been synthesized and characterized by elemental analyses, spectral (UV-Vis, IR, ^1^H NMR, Fluorescence, and ESR) studies, thermal techniques, and magnetic moment measurements. The heterocyclic Schiff bases act as bidentate ligands and coordinate with metal ions through nitrogen and sulphur of the thiol group. The low molar conductance values in DMF indicate that the metal complexes are nonelectrolytes. The magnetic moments and electronic spectral data suggest octahedral geometry for the Co(II), Ni(II), and Zn(II) complexes and square planar for Cu(II) complexes. Two Gram-positive bacteria (*Staphylococcus aureus* MTCC 96 and *Bacillus subtilis* MTCC 121), two Gram-negative bacteria (*Escherichia coli* MTCC 1652 and *Pseudomonas aeruginosa* MTCC 741), and one yeast, *Candida albicans*, were used for the evaluation of antimicrobial activity of the newly synthesized compounds.

## 1. Introduction

Recently, for the rapid development of drug resistance, new antimicrobial agent should be designed and synthesized with chemical characteristics clearly different from those of existing ones. 1,2,4-triazoles and their fused heterocyclic derivatives have received considerable attention owing to their synthetic and effective biological importance such as analgesic [1], antitumor [2], anticancer [3, 4], antimicrobial [5–7], anticonvulsant [8, 9], and antiproliferative activities [10]. In addition, 1,2,4-triazole and, in particular, its derivatives exhibit a strong property of acting as a bridging ligand between two metal centers [11] and can provide 1,2-bridiging as well as 2,4-bridiging form in case of 4-unsubstituted 1,2,4-triazoles.

Schiff bases represent an important class of compounds because they are utilized as starting materials in the synthesis of industrial products [12]. Schiff bases derived from 3-substituted-4-amino-5-mercapto-1,2,4-triazoles show analgesic, antimicrobial, anti-inflammatory, and antidepressant activities [13]. When bioorganic molecules or drugs are bound to metal ions, there is a drastic change in their biomimetic properties, therapeutic effects, and pharmacological properties. Metal complexes of Schiff bases derived from triazoles have been synthesized, and it has been observed that antimicrobial activity of the Schiff bases is significantly enhanced when coordinated to metal ions [14, 15]. The metal complexes containing substituted 1,2,4-triazole ligands have spin-crossover properties and can be used as molecular-based memory devices, display and optical switches [16, 17].

Keeping in view medicinal and industrial applications of triazoles and potential chemistry of transition metals; new heterocyclic Schiff bases of 4-amino-5-mercapto-3-H/propyl-1,2,4-triazole with 5-nitrofurfuraldehyde [HL^1-2^] and their metal complex with Co(II), Ni(II), Cu(II), and Zn(II) ions have been synthesized. The synthesized ligands and their metal complexes were characterized by different techniques like elemental analyses, spectral studies (UV-Vis, IR, ^1^H NMR, Fluorescence, ESR), TGA, and magnetic and conductance measurements. The newly synthesized compounds were evaluated for their antimicrobial studies.

## 2. Experimental

### 2.1. Materials

All starting precursor were of analytical grade. The reagents and solvents were purchased commercially and used without further purification unless otherwise noted. 4-Amino-5-mercapto-1,2,4-triazole (AMT) and 4-amino-5-mercapto-3-propyl-1,2,4-triazole (AMPT) were prepared by reported literature method [18].

### 2.2. Syntheses of Schiff Bases

#### 2.2.1. 4-((5-Nitrofuran-2-yl)methyleneamino)-5-mercapto-1,2,4-triazole (HL^1^)

 4-Amino-5-mercapto-1,2,4-triazole (0.61 g, 5.06 mmol) dissolved in warm ethanol (20 mL) was added to an ethanol solution (30 mL) containing 5-nitrofurfuraldehyde (0.72 g, 5.06 mmol). The mixture was refluxed for 5 hrs. The reaction mixture was then cooled to room temperature and the yellow solid formed was filtered. It was then recrystallized from ethanol and dried.

 m.p. 168–170°C, (Found: C, 35.01; H, 2.11; N, 29.28%. Calcd. for C_7_H_5_N_5_O_3_S: C, 35.15; H, 2.11; N, 29.28%).

#### 2.2.2. 4-((5-Nitrofuran-2-yl)methyleneamino)-5-mercapto-3-propyl-1,2,4-triazole (HL^2^)

To a solution of 5-nitrofurfuraldehyde (0.52 g, 3.68 mmol) in ethanol (30 mL), 4-amino-5-mercapto-3-propyl-1,2,4-triazole (0.58 g, 3.68 mmol) in ethanol (20 mL) was added. The mixture was refluxed for 6 h. The reaction mixture was then cooled to room temperature and the reddish yellow solid formed was filtered. It was then recrystallized from ethanol and dried.

 m.p. 142–144°C, (Found: C, 42.70; H, 3.33; N, 24.33%. Calcd. for C_10_H_11_N_5_O_3_S: C, 42.70; H, 3.94; N, 24.90%).

### 2.3. Syntheses of Metal Complexes

#### 2.3.1. Metal Complexes of 4-((5-Nitrofuran-2-yl)methyleneamino)-5-mercapto-1,2,4-triazole (HL^1^)

The metal complexes were synthesized by reacting aqueous ethanolic solutions of acetates of Co(II) (0.14 g, 0.57 mmol), Ni(II) (0.14 g, 0.57 mmol), Cu(II) (0.11 g, 0.57 mmol), and Zn(II) (0.13 g, 0.57 mmol) with the hot ethanolic solutions of the HL^1^ (0.27 g, 1.14 mmol). The solid complexes formed were filtered off and washed several times with warm water, aqueous ethanol to remove unreacted metal acetates or ligands, and finally with acetone and vacuo dried.

 Co(L^1^)_2_·2H_2_O: (Found: C, 29.21; H, 2.01; N, 24.11; Co, 10.13% Calcd. for C_14_H_12_CoN_10_O_8_S_2_: C, 29.43; H, 2.12; N, 24.51; Co, 10.31%).
 Ni(L^1^)_2_·2H_2_O: (Found: C, 29.12; H, 2.02; N, 24.08; Ni, 10.11% Calcd. for C_14_H_12_N_10_NiO_8_S_2_: C, 29.44; H, 2.12; N, 24.52; Ni, 10.28%).
 Cu(L^1^)_2_: (Found: C, 31.00; H, 1.39; N, 25.88; Cu, 11.40% Calcd. for C_14_H_8_CuN_10_O_6_S_2_: C, 31.14; H, 1.49; N, 25.94; Cu, 11.77%).
 Zn(L^1^)_2_·2H_2_O: (Found: C, 28.96; H, 1.99; N, 24.20; Zn, 11.31% Calcd. for C_14_H_12_N_10_O_8_S_2_Zn: C, 29.10; H, 2.09; N, 24.24; Zn, 11.32%).

#### 2.3.2. Metal Complexes of 4-((5-Nitrofuran-2-yl)methyleneamino)-5-mercapto-3-propyl-1,2,4-triazole (HL^2^)

The metal acetates of Co(II) (0.17 g, 0.67 mmol), Ni(II) (0.17 g, 0.67 mmol), Cu(II) (0.13 g, 0.67 mmol), and Zn(II) (0.15 g, 0.67 mmol) in aqueous ethanol were treated with hot ethanolic solution of the HL^2^ (0.37 g, 1.34 mmol). The colored complexes formed were filtered off and washed several times with warm water, aqueous ethanol to remove unreacted metal acetates or ligands, and finally with acetone and vacuo dried.

 Co(L^2^)_2_·2H_2_O: (Found: C, 36.22; H, 3.47; N, 21.23; Co, 8.23% Calcd. for C_20_H_24_CoN_10_O_7_S_2_: C, 36.64; H, 3.69; N, 21.37; Co, 8.99%).
 Ni(L^2^)_2_·2H_2_O: (Found: C, 36.53; H, 3.51; N, 21.18; Ni, 8.53% Calcd. for C_20_H_24_N_10_NiO_7_S_2_: C, 36.66; H, 3.69; N, 21.37; Ni, 8.96%).
 Cu(L^2^)_2_: (Found: C, 38.15; H, 3.18; N, 22.13; Cu, 10.23% Calcd. for C_20_H_20_CuN_10_O_6_S_2_: C, 38.49; H, 3.23; N, 22.44; Cu, 10.18%).
 Zn(L^2^)_2_·2H_2_O: (Found: C, 36.06; H, 3.43; N, 21.15; Zn, 9.43% Calcd. for C_20_H_24_N_10_O_7_S_2_Zn: C, 36.29; H, 3.65; N, 21.16; Zn, 9.88%).

### 2.4. Analyses and Instrumentation

Elemental analyses (C, H, and N) were performed on Perkin-Elmer 2400 Elemental Analyzer available at SAIF, Punjab University, Chandigarh. The metal contents were determined gravimetrically by a literature procedure [19] after digesting the organic matter with aqua regia and evaporating the residue to dryness. IR spectra (4000–250 cm^−1^) of the ligands and their metal complexes were recorded on an MB-3000 ABB Spectrometer. The electronic absorption spectra were recorded on T 90 (PG Instruments ltd) UV/Vis spectrometer in the region 1100–200 nm. ^1^H NMR spectra were recorded in DMSO-d_6_ on a Bruker ACF 300 spectrometer at 300 MHz using “tetramethyl silane” as the internal standard. Magnetic moment measurements were carried out at room temperature on vibrating sample magnetometer (Model 155) at Institute Instrumentation Centre, IIT Roorkee. The Perkin Elmer (Pyris Diamond) instrument was used to carry out thermal analysis of metal complex in atmospheric air (50–800°C) at a heating rate of 10°C min^−1^ using a reference to alumina powder. The fluorescence studies of Schiff bases and their metal complexes were recorded on SHIMADZU RF-5301PC spectrophotometer. The solutions of 10^-3 ^M concentration were prepared in HPLC-grade DMF, and the experiment was carried out at room temperature. ESR spectra were recorded on X-Band at frequency of 9.1 GHz under the magnetic field 3000 Guass on a varian E-112 ESR spectrometer at SAIF, IIT Bombay.

### 2.5. Antimicrobial Assay

#### 2.5.1. Test Microorganisms

Two Gram-positive bacteria (*Staphylococcus aureus* MTCC 96 and *Bacillus subtilis* MTCC 121), two Gram-negative bacteria (*Escherichia coli* MTCC 1652 and *Pseudomonas aeruginosa* MTCC 741), and one Yeast *Candida albicans* were used in the present study for evaluation of antimicrobial activity of the synthesized compounds. All the bacterial cultures were procured from Microbial Type Culture Collection (MTCC), IMTECH-Chandigarh. Medium used for the antimicrobial testing was Muller Hilton agar media and autoclaved at 15 lbs/in^2^ for 15 min.

#### 2.5.2. Antimicrobial Activity

The antimicrobial activity of the newly synthesized compounds was assayed by using agar wells diffusion technique [20, 21]. For the evaluation of antimicrobial activity, the size of inoculum was adjusted to approximately 10^8^ colony-forming units (cfu/mL) by suspending the culture in sterile distilled water. Petri dishes containing 20 mL of Muller Hilton agar medium were swabbed with a culture of the respective microbial strains and kept for 15 min for the absorption of culture. Sterile borer is used to create the wells (6 mm in diameter), and we added 100 *μ*L solution of each compound of 4.0 mg/mL concentration reconstituted in the DMSO on the preinoculated plates. All the plates were incubated at 37°C for 24 hrs. Antimicrobial activity of all the synthesized compounds was determined by measuring the zone of inhibition around the wells. DMSO was used as a negative control, whereas Ciprofloxacin was used as positive control. This procedure was performed in three replicate plates for each organism.

#### 2.5.3. Determination of Minimum Inhibitory Concentration (MIC)

MIC of all the compounds was determined by the modified agar well diffusion method [22]. Different concentrations ranging from 10 to 1000 *μ*g/mL of synthesized compounds were made from the stock solution of 4 mg/mL in DMSO. A 100 *μ*L volume of each dilution was introduced into wells (in triplicate) in the agar plates already seeded with 100 *μ*L of standardized inoculum (10^8^ cfu/mL) of the test microbial strain. These plates were incubated at 37°C for 24 h and observed for the inhibition zones. Ciprofloxacin antibiotic was taken as positive control.

## 3. Results and Discussion

**Figure 1:**
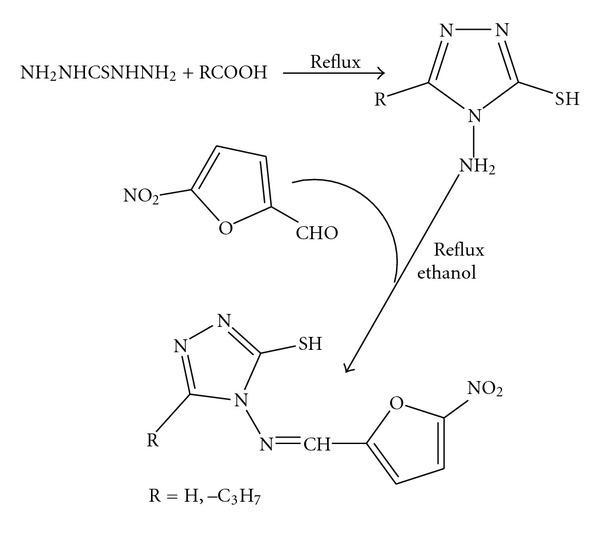
Scheme for the syntheses of Schiff bases.

The scheme for the syntheses of Schiff bases is represented in Figure 1. The complexes are soluble in DMF and DMSO and are insoluble in common organic solvents. The elemental analyses data show that the metal to ligand ratio is 1 : 2 in all the complexes. The complexes are powdery solids, colored, and nonhygroscopic in nature. The molar conductance values of the complexes (measured in 10^-3 ^M DMF) at room temperature lie in the range 6.8–11.3 ohm^-1 ^cm^2 ^mol^−1^, which suggest that they are nonelectrolytes [23, 24]. The purity of ligands and their metal complexes has been checked by TLC.

### 3.1. ^1^H NMR and ^13^C NMR Spectra

The ^1^H NMR spectral data of Schiff bases (HL^1-2^) and their Zn(II) complexes have been given in Table 1 [14, 15, 25]. In the spectra of free ligands, signals observed at *δ* 14.10 (HL^1^) and 13.91 (HL^2^) can be assigned to the SH protons. These signals disappeared in the spectra of metal complexes, which confirms the coordination of ligand to metal ion through the deprotonated thiol group. In the spectra of Schiff bases, signals at *δ* 9.86 and 10.42 are assigned to azomethine protons. The characteristic signal due to azomethine proton shifted downfield in the spectra of metal complexes indicating coordination through the azomethine nitrogen. The aromatic protons present in the ligands (HL^1-2^) are found in the region *δ* 7.55–7.84 ppm. The triazole-H of HL^1^ appeared as singlet at 8.97 ppm and propyl group protons of HL^2^ Schiff base appeared as triplet *δ* 0.93 (–CH_3_), triplet *δ* 2.69 (–CH_2_–), and multiplet at *δ* 1.66 (–CH_2_–) ppm. These protons signals of Schiff bases show a slight shift upon coordination with metal ions.

The ^13^C NMR spectral data of Schiff bases (HL^1-2^) and their Zn(II) complexes have been given in Table 1. Schiff bases show signal at *δ* 163.34 (HL^1^) and 161.9 (HL^2^) for their azomethine carbons, and they shift downfield in their corresponding zinc(II) complexes due to the coordination through azomethine nitrogen. 

### 3.2. IR Spectra

The bonding of the ligands to metal ions has been judged by careful comparison of the infrared spectra of the complexes with those of the free ligand (Table 2). Some important bands have been selected to observe the effect on ligand vibration in the complexes. The formation of ligand is confirmed by the absence of stretching vibrations due to aldehyde *ν*(CHO) and amino *ν*(NH_2_) moiety of triazole and instead of this a strong new band appeared at 1622–1625 cm^−1^ corresponding to the azomethine *ν*(HC=N) group. After complexation, the band due azomethine vibration shifted to lower frequency (10–15 cm^−1^), thus indicating the coordination of the azomethine-N to metal ions [26]. The ligands show a characteristic strong band at 2739–2770 cm^−1^, which is attributed to *ν*(SH), disappeared in the spectra of metal complexes, confirming deprotonation and coordination of thiol group [27]. This is further supported by the lower frequency band appeared at 710–735 cm^−1^ in the metal complexes due to *ν*(C–S). A broad band in the region 3355–3445 cm^−1^ assigned as *ν*(OH) indicates the presence of water molecules in the complexes. The metal ligand bands appear in the region 347–362 cm^−1^ and 490–515 cm^−1^ in all the complexes that have been assigned to *ν*(M–S) and *ν*(M–N), respectively [24, 28].

### 3.3. ESR Spectra

**Figure 2:**
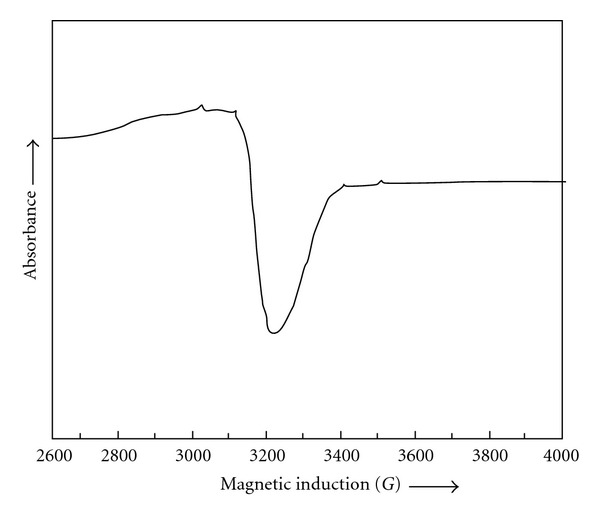
X-Band ESR spectrum of Cu(L^1^)_2_.

The electronic paramagnetic resonance spectra of Cu(L^1^)_2_ (Figure 2) and Cu(L^2^)_2_ were recorded in solid state at room temperature to obtain further information about their stereochemistry. The *g*-tensor values of the Cu(L^1^)_2_ (*g*_||_ = 2.12, *g*_⊥_ = 2.05, *g*_av_ = 2.07, *G* = 2.46) and Cu(L^2^)_2_ (*g*_||_ = 2.13, *g*_⊥_ = 2.05, *g*_av_ = 2.07, *G* = 2.66) complexes can be used to derive the ground state. In square planar complexes, the unpaired electron lies in *dx* ^2^-*y* ^2^ orbital giving ^2^ *B*_1*g*_ as the ground state with *g*_||_ > *g*_⊥_ > 2, while the unpaired electron lies in the *dz* ^2^ orbital giving ^2^ *A*_1*g*_ as the ground state with *g*_⊥_ > *g*_||_ > 2. In the present case, *g*_||_ > *g*_⊥_ > 2, therefore the unpaired electron is likely to be in the *dx* ^2^-*y* ^2^ orbital, indicating square planar geometry around the copper(II) ion [29, 30]. No signal at half field was observed in the spectrum, ruling out the possibility of a dimeric form [31].

### 3.4. Magnetic Moment Measurements and Electronic Spectra

The observed electronic transitions and calculated ligand field parameters of the metal complexes are listed in Table 3. The electronic spectra provided enough information regarding the arrangements of the ligands around the metal ions. Co(II) complexes of HL^1-2^ at room temperature show magnetic moments 4.11 and 4.20 BM, respectively [25, 27]. These values are in good agreement with those reported for octahedral Co(II) complexes. The Co(II) complexes exhibited two distinct absorptions in the regions 10599–10913 cm^−1^ and 20809–20998 cm^−1^ assigned to ^4^T_1*g*_(F) → ^4^T_2*g*_(F) (*ν*_1_) and ^4^T_1*g*_(F) → ^4^T_1*g*_(P) (*ν*_3_) transitions, respectively, which suggests octahedral geometry around the Co(II) ion [14, 25, 27]. *ν*_2_ is not observed, but it is calculated by using relation *ν*_2_ = *ν*_1_ + 10*Dq*, which is very close to (*ν*_3_) transition [32]. 

The Ni(II) complex reported herein are high spin with room temperature magnetic moment value ~3.75 BM, which is in the normal range observed for octahedral complexes. The electronic spectra of Ni(II) complexes displayed three bands in the regions 9907–9933 cm^−1^, 16226–16283 cm^−1^, and 23415–24372 cm^−1^, assigned to ^3^ *A*_2*g*_(F)  → ^3^T_2*g*_(F) (*ν*_1_), ^3^ *A*_2*g*_(F)  → ^3^T_1*g*_(F) (*ν*_2_), and ^3^ *A*_2*g*_(F)  → ^3^T_1*g*_(P) (*ν*_3_) transitions, respectively [24–26]. These are the characteristic bands of octahedral environment around Ni(II) ion.

The Band-fitting equations [32, 33] have been used to calculate the ligand field parameters (*Dq*, *B*, *β*, and *β*%) for Co(II) and Ni(II) complexes indicated significant covalent character of metal ligand bonds (Table 3). The value of Rachah parameter (*B*) is less than free ion value, suggesting an orbital overlap and delocalization of electron on the metal ion. The nephelauxetic ratio (*β*) for the metal complexes is less than one suggesting partial covalency in the metal ligand bond.

The copper complexes of HL^1-2^ Schiff bases show band at 17981 and 18121 cm^−1^, respectively, which can be assigned to ^2^ *B*_1*g*_ → ^2^ *A*_1*g*_ (*ν*_1_) transition. It is a characteristic band of square planar geometry around the Cu(II) [25, 34]. The room temperature magnetic moment value 1.86–1.92 BM falls in the range normally observed for square planar complexes. 

### 3.5. Fluorescence Spectra

**Figure 3:**
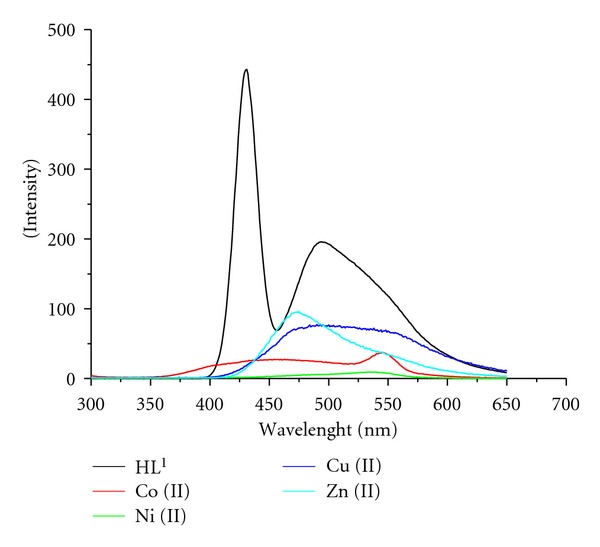
Fluorescence spectra of HL^1^ and its metal complexes.

**Figure 4:**
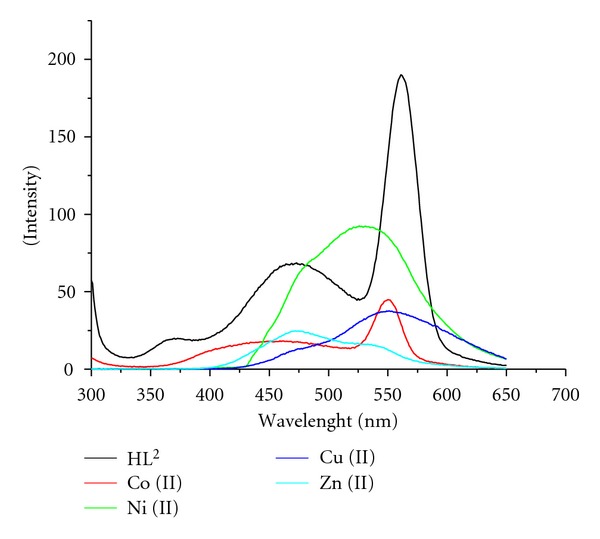
Fluorescence spectra of HL^2^ and its metal complexes.

In order to investigate the effect of M(II) ions on the fluorescence of the ligands, the fluorescence spectra of ligands (HL^1-2^) and their metal complexes have been recorded in 10^-3 ^M DMF solution. The overlapping spectra of the ligands and their Co(II), Ni(II), Cu(II), and Zn(II) complexes are given in Figures 3 and 4. HL^1^ exhibits two strong fluorescence emission bands at 431 and 494 nm (Ex 280 nm), and its metal complexes show fluorescence emission bands at 463, 545 nm for Co(II), 403, 536 nm for Ni(II), 493 nm for Cu(II), and 475 nm for Zn(II). HL^2^ shows one weak emission band at 370 nm and two strong fluorescence emission bands at 473 and 561 nm (Ex. 275 nm), while its metal complexes show emission bands at 451, 550 nm for Co(II), 526 nm for Ni(II), 551 nm for Cu(II), and 475 nm for Zn(II). It has been observed from the fluorescence emission spectra that transition metal ions decrease the fluorescence intensity of free ligands. The results of our study are in accordance with the reports of earlier workers [26, 35], which also observed partial fluorescence quenching phenomena in the metal complexes. Magnetic perturbation, redox activity, and so forth. have been invoked in the past to rationalize fluorescence quenching by transition metal ions [36].

### 3.6. Thermal Studies

**Figure 5:**
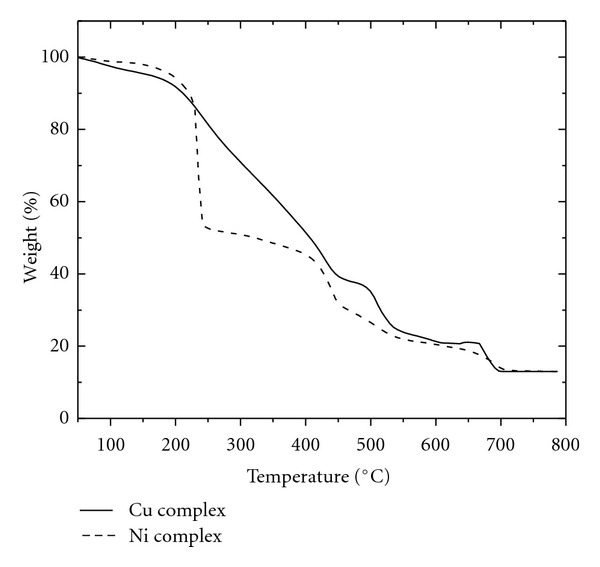
Thermogravimetric Curves of Ni(L^1^)_2_·2H_2_O and Cu(L^1^)_2_ complexes.

Thermogravimetric analyses of the complexes Ni(L^1^)_2_·2H_2_O and Cu(L^1^)_2_ are given in Table 4. The correlations between the different decomposition steps of the complexes with their corresponding mass losses are discussed in terms of the proposed formulae of the complexes. The results show good agreement with the formulae suggested from the analytical data. The TG curve of Ni(L^1^)_2_·2H_2_O (Figure 5) consists mainly of three steps in the temperature range 50–190, 190–424, and 424–750°C. The first step seems to be consistent with the evolution of two water molecules (calcd. 6.3%, found 6.9%). The second TG step represents a mass loss (calcd. 48.6%, found 48.2%) corresponding to the removal of organic moiety [C_10_H_6_N_4_O_6_]. The final step corresponds to decomposition of triazole molecules at 424–750°C with mass loss of 34.5% (calcd. 34.6%) of the ligand leaving metal oxide as residue. The TG curve of the Cu(L^1^)_2_ complex shows three stages of decomposition within the temperature range 50–750°C. The first stage at 50–245°C correspond to loss of organic moiety with mass loss of 46.9% (calcd. 46.3%). The second and third steps corresponds to removal of two triazole molecules in the temperature range 245–750°C with a total mass loss of 40.4% (calcd. 41.8%). The decomposition of all the complexes ended with oxide formation [24, 25, 27].

### 3.7. *In Vitro* Antimicrobial Discussion

The ligands (HL^1-2^), metal complexes, standard drugs, and DMSO solvent were screened separately for their antimicrobial activity against Gram-positive (*Staphylococcus aureus, Bacillus subtilis*) and Gram-negative (*Escherichia coli, Pseudomonas aeruginosa*) bacteria and against yeast *Candida albicans. *The microbial results are summarized in Tables 5 and 6. The comparative studies of the Schiff bases and their metal complexes indicate that the complexes showed significantly enhanced antimicrobial activity against microbial strains in comparison to the free ligands. Positive controls (Standard drug) produced significantly sized inhibition zones against the tested bacteria; however, negative control (DMSO) produced no observable inhibitory effect against any of the test organisms.

The newly synthesized compounds showed zone of inhibition ranging from 12.6 mm to 19.6 mm against the Gram positive bacteria and 12.7 mm to 21.2 mm against Gram negative bacteria. On the basis of zone of inhibition produced against the test bacterium, it is observed that Cu(L^1^)_2_ shows antibacterial activity comparable to standard drug in case of *Pseudomonas aeruginosa*. However, some of the compounds in this series were not effective against some bacterial strains. All the compounds showed good antimicrobial activity against *Candida albicans* ranging from 14.6 mm to 19.5 mm each. Co(L^1^)_2_·2H_2_O and Cu(L^1^)_2_ were found to be most effective against *Candida albicans *with zone of inhibition of 19.5 mm. MIC results also revealed that the metal complexes are slightly more effective against the antimicrobial strains as compared to the Schiff bases. Co(L^1^)_2_·2H_2_O was found to be the best antimicrobial agent exhibited the lowest MIC 100 *μ*g/mL against *Bacillus subtilis *and* Candida albicans* (Table 6). The results of our study are in accordance with the reports of earlier workers [25, 26], which also showed that the antimicrobial activity of ligands is greatly enhanced when it is coordinated to metal ions.

The overtone's concept [37] and Tweedy's chelation theory [38] can be used to explain the enhanced in antimicrobial activity of the metal complexes. According to the Overtone's concept of cell permeability, the lipid membrane surrounding the cell favors the passage of only lipid-soluble materials; therefore, liposolubility is an important factor which controls the antimicrobial activity. On chelation, polarity of the metal ion is reduced to a greater extent due the overlapping of the ligand orbital and partial sharing of the positive charge of the metal ion with donor groups. Moreover, delocalization of the *π*-electrons over the whole chelate ring is increased, and lipophilicity of the complexes is enhanced. The increased lipophilicity enhances the penetration of the complexes into the lipid membranes and blocks the metal binding sites in the enzymes of microorganisms. These complexes also disturb the respiration process of the cell and thus block the synthesis of proteins, which restricts further growth of the organism. In general, metal complexes are more active than ligands as they may serve as principal cytotoxic species.

## 4. Conclusions

**Figure 6:**
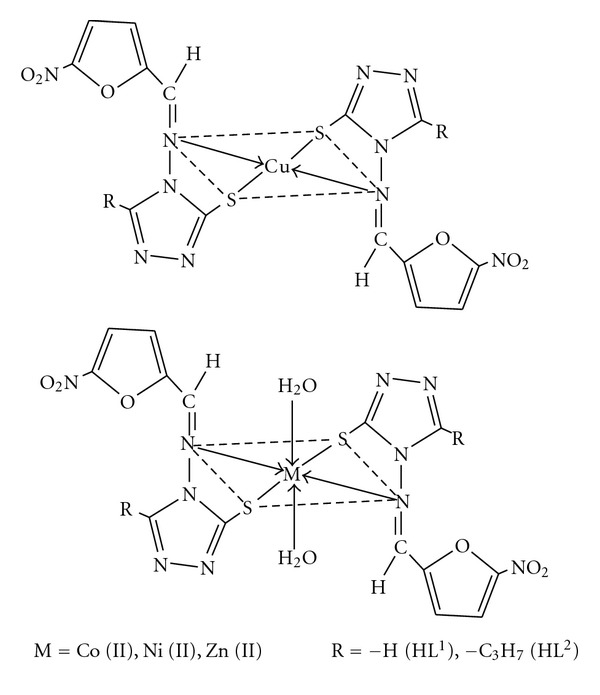
Structures of Metal Complexes.

The synthesized Schiff bases act as bidentate ligands and coordinated to metal ion through azomethine nitrogen and sulphur of thiol group. The bonding of ligand to metal ion is confirmed by elemental analyses, spectral studies (UV-Vis, IR, ^1^H NMR, ESR, Fluorescence), TGA, and magnetic and conductance measurements. The spectral studies suggested octahedral geometry for the Co(II), Ni(II), and Zn(II) complexes and square planar for Cu(II) complexes (Figure 6). No signal at half field was observed in the ESR spectrum, ruling out the possibility of a dimeric form. The antimicrobial studies suggested that the Schiff bases were found to be biologically active and their metal complexes show significantly enhanced antimicrobial activity against microbial strains in comparison to the free ligands. Thus, exhibiting their broad spectrum nature can be further used in pharmaceutical industry for mankind, as an antimicrobial agent, after testing its toxicity to human beings.

## Figures and Tables

**Table 1:** ^
1^H NMR and ^13^C NMR spectral data of Schiff bases and their metal complexes.

Compounds	^ 1^H NMR (DMSO-d_6_) (ppm)	^ 13^C NMR (DMSO-d_6_) (ppm)
HL^1^ [C_7_H_5_N_5_O_3_S]	7.55 (d, 1H, Ar–H), 7.84 (d, 1H, Ar–H), 8.97 (s, triazole–H), 9.86 (s, 1H, –N=CH–), 14.10 (s, 1H, –SH)	114.37, 120.34, 139.44, 146.98, 149.43, 152.12 (Aromatic), 163.34 (–N=CH–)
Zn(L^1^)_2_·2H_2_O [C_14_H_12_N_10_O_8_S_2_Zn]	7.42 (d, 2H, Ar–H), 7.66 (d, 2H, Ar–H), 9.05 (s, 2H, triazole–H), 10.12 (s, 2H, –N=CH–)	114.31, 121.02, 139.28, 146.76, 150.22, 152.38 (Aromatic), 171.18 (–N=CH–)
HL^2^ [C_10_H_11_N_5_O_3_S]	0.93 (t, 3H, –CH_3_), 1.66 (m, 2H, –CH_2_–), 2.69 (t, 2H, –CH_2_–), 7.63 (d, 1H, Ar–H), 7.84 (d, 1H, Ar–H), 10.42 (s, 1H, –N=CH–), 13.91 (s, 1H, –SH)	13.84 (–CH_3_), 19.21 (–CH_2_–), 26.69 (–CH_2_–), 114.32, 120.4, 148.28, 149.50, 151.97, 153.52 (Aromatic), 161.9 (–N=CH–)
Zn(L^2^)_2_·2H_2_O [C_20_H_24_N_10_O_7_S_2_Zn]	0.80 (t, 6H, –CH_3_), 1.57 (m, 4H, –CH_2_–), 2.63 (t, 4H, –CH_2_–), 7.69 (d, 2H, Ar–H), 7.85 (d, 2H, Ar–H), 10.83 (s, 2H, –N=CH–)	13.74 (–CH_3_), 19.63 (–CH_2_–), 26.79 (–CH_2_–), 114.22, 121.5, 148.75, 152.87, 153.50, 153.70 (Aromatic), 169.72 (–N=CH–)

**Table 2:** Important IR spectral bands (cm^−1^) of Schiff bases and their metal complexes.

Compound	*ν*(N=CH)	*ν*(C–S)	*ν*(S–H)	*ν*(H_2_O/OH)	*ν*(M–S)	*ν*(M–N)
HL^1^	1622	—	2739	—	—	—
Co(L^1^)_2_·2H_2_O	1613	733	—	3441	360	502
Ni(L^1^)_2_·2H_2_O	1607	734	—	3356	347	512
Cu(L^1^)_2_	1612	715	—	—	351	497
Zn(L^1^)_2_·2H_2_O	1610	717	—	3425	361	511
HL^2^	1625	—	2770	—	—	—
Co(L^2^)_2_·2H_2_O	1613	735	—	3440	355	493
Ni(L^2^)_2_·2H_2_O	1611	730	—	3410	347	490
Cu(L^2^)_2_	1609	710	—	—	362	503
Zn(L^2^)_2_·2H_2_O	1606	718	—	3395	353	510

**Table 3:** Electronic spectral data (in DMF) and ligand field parameters of metal complexes.

Compound	Transitions (cm^−1^)			*Dq* (cm^−1^)	*B* (cm^−1^)	*ν*_2_/*ν*_1_	*β*	*β*%	*μ*_eff_ (BM)
	*ν*_1_	*ν*_2_	*ν*_3_						
Co(L^1^)_2_·2H_2_O	10599	22425*	20809	1182.6	762.4	2.11	0.78	22	4.20
Co(L^2^)_2_·2H_2_O	10913	23063*	20998	1215.0	754.8	2.11	0.77	22	4.11
Ni(L^1^)_2_·2H_2_O	9907	16226	24372	990.7	725.1	1.63	0.69	31	3.79
Ni(L^2^)_2_·2H_2_O	9933	16283	23415	993.3	659.9	1.63	0.63	37	3.73
Cu(L^1^)_2_	17981			—	—	—	—	—	1.86
Cu(L^2^)_2_	18121			—	—	—	—	—	1.92

*Calculated value.

**Table 4:** Thermogravimetric data of Ni(L^1^)_2_·2H_2_O and Cu(L^1^)_2_ complexes.

Compound	Decomposition stages and assignment	Temp. (°C)	% Weight loss found (Calcd.)
Ni(L^1^)_2_·2H_2_O [C_14_H_12_N_10_NiO_8_S_2_]	(1) Water molecules	50–190	6.9 (6.3)
	(2) Organic moiety	190–424	48.2 (48.6)
	(3) Triazoles moiety	424–750	34.5 (34.6)

Cu(L^2^)_2_ [C_14_H_8_CuN_10_O_6_S_2_]	(1) Organic moiety	50–245	46.9 (46.3)
	(2) Triazole moiety	245–445	20.1 (20.9)
	(3) Triazole moiety	445–750	20.3 (20.9)

**Table 5:** Antimicrobial activity of the synthesized compounds.

Compound	Diameter of growth of inhibition zone (mm)				
	*Staphylococcus aureus *	*Bacillus subtilis *	*Escherichia coli *	*Pseudomonas aeruginosa *	*Candida albicans *
HL^1^	17.3	19.6	15.2	12.7	15.2
Co(L^1^)_2_·2H_2_O	17.4	16.5	16.3	—	19.5
Ni(L^1^)_2_·2H_2_O	18.2	16.9	15.4	—	16.5
Cu(L^1^)_2_	17.2	18.4	17.6	21.2	19.5
Zn(L^1^)_2_·2H_2_O	15.4	16.8	17.4	17.6	15.8
HL^2^	15.4	12.6	15.4	13.7	15.3
Co(L^2^)_2_·2H_2_O	—	14.6	19.4	17.3	14.8
Ni(L^2^)_2_·2H_2_O	—	14.8	16.2	17.3	17.9
Cu(L^2^)_2_	—	—	19.2	17.4	14.6
Zn(L^2^)_2_·2H_2_O	15.4	—	13.3	12.4	15.7
Ciprofloxacin	23.0	24.0	23.0	20.0	nt

—: Indicates no activity, nt: not tested.

**Table 6:** Minimum inhibitory concentration (MIC) (*μ*g/mL) of synthesized compounds.

Compounds	*Staphylococcus aureus *	*Bacillus subtilis *	*Escherichia coli *	*Pseudomonas aeruginosa *	*Candida albicans *
NFMT	300	200	400	800	200
Co(L^1^)_2_·2H_2_O	300	100	200	—	100
Ni(L^1^)_2_·2H_2_O	100	400	200	—	200
Cu(L^1^)_2_	200	200	200	500	200
Zn(L^1^)_2_·2H_2_O	400	500	500	700	500
NFMPT	500	800	300	500	700
Co(L^2^)_2_·2H_2_O	—	800	500	700	700
Ni(L^2^)_2_·2H_2_O	—	500	800	400	700
Cu(L^2^)_2_	—	—	100	600	500
Zn(L^2^)_2_·2H_2_O	200	—	500	800	200
Ciprofloxacin	5	5	5	5	—

—: Not tested.
